# Economic choice between remifentanil and food in squirrel monkeys

**DOI:** 10.1038/s41386-021-00996-6

**Published:** 2021-04-08

**Authors:** Samantha O. Brown, Devin P. Effinger, Rodrigo A. Montoro, Nabil Daddaoua, Zuzana Justinova, Megan J. Moerke, Charles W. Schindler, Hank P. Jedema, Charles W. Bradberry

**Affiliations:** Intramural Research Program, National Institute on Drug Abuse, Baltimore, MD USA

**Keywords:** Addiction, Decision

## Abstract

Traditional approaches for evaluating if compounds are reinforcing, and thus a risk for abuse, include preclinical self-administration procedures conducted in the absence of alternative reinforcers. While the track record of this approach for determining abuse potential is good, that for predicting efficacy of addiction treatments is not. An alternate approach would be economic choice between drug and nondrug rewards, with parametrically varied options from trial to trial. This would promote goal-directed decisions between reward modalities and should provide metrics that reflect changes in internal state that influence desirability of a given option. We report herein a high throughput economic choice procedure in which squirrel monkeys choose between a short-lived opiate, remifentanil, and a palatable food reward. Stimuli on touchscreens indicate the amount of each reward type offered by varying the number of reward-specific elements. The rapid clearance of remifentanil avoids accumulation of confounding levels of drug, and permits a large number of trials with a wide range of offers of each reward modality. The use of a single metric encompassing multiple values of each reward type within a session enables estimation of indifference values using logistic regression. This indifference value is sensitive to reward devaluation within each reward domain, and is therefore a useful metric for determining shifts in reward preference, as shown with satiation and pharmacological treatment approaches.

## Introduction

Self-administration studies have long served as the cornerstone of preclinical research for understanding drug reinforcement and searching for improved addiction therapies. Traditionally, the self-administration procedure has utilized rate-dependent measures of drug consumption, in the absence of alternative reinforcers. A behavioral approach with real-world parallels is one in which the consumption of drugs comes at the expense of alternative nondrug rewards, given that the choice to use drugs can result in the loss of a job, good family relations, and/or good health [1–3]. In human laboratory studies, cocaine users choose sufficiently high monetary rewards over the option to self-administer cocaine [4], demonstrating that drug users are able to minimize their drug intake when behavioral alternatives are available. Contingency management, the therapeutic extension of this, is an effective nonpharmacological approach for the treatment of substance use disorders [5].

Behavioral economic theory and concurrent choice procedures in clinical settings have provided a framework for evaluating how individual variability in severity of addiction relates to distinct factors that could influence subjective valuation as reflected in choice between drug and nondrug rewards. The relationships between addiction severity or recovery success with differences in sensitivity to the effect of drug-associated stimuli, drug devaluation, or punishment on drug choice, have enabled conceptual conclusions about mechanisms of vulnerability and treatment efficacy [6]. Though addiction severity or treatment success is not being modeled herein, those clinical applications highlight the value of an economic decision-making approach.

Nonhuman primate studies using macaques benefit from the wealth of their prior use in studies of the behavioral pharmacology of drug reward and electrophysiological studies of neuroeconomics and reward-driven choice. However, the complexities and costs of working with macaques biases approaches toward within-subject evaluation in a small number of individual subjects. While rodent models are well-suited for between-group comparisons and the use of novel tools, squirrel monkeys could potentially provide a compromise approach with increased statistical power using nonhuman primate subjects. A statistically well-powered sample demonstrating consistent effects between groups of subjects increases the potential to explore novel addiction therapies in nonhuman primates, which would benefit from their closer proximity and similarity to humans [7, 8]. To establish a broad exploratory platform for these approaches, we report herein the results of work establishing a squirrel monkey model of economic choice between the short-lived opioid remifentanil [9, 10] and a palatable food reward, diluted sweetened condensed milk [11]. Using a touchscreen apparatus, we trained squirrel monkeys to complete a choice task in which the magnitude of the remifentanil and sweetened condensed milk rewards offered were varied from trial to trial and evaluated the effects of pharmacological manipulations in terms of choice allocation, rather than response rates wherein alterations can reflect nonspecific motoric and/or cognitive effects [12].

The aims of the present study were to: (1) establish a behavioral choice paradigm that affords a large number of trials for statistical power and a wider parametric range of choices; (2) demonstrate the economic nature of choices made by the subjects; (3) evaluate the impact of pharmacological manipulation of remifentanil reward on choice behavior by pretreating animals with a μ-opioid receptor agonist and antagonist.

## Materials and methods

### Subjects

The present study includes ten adult male squirrel monkeys (Saimiri sciureus; BW 700-1100 g) without previous touchscreen experience. Monkeys were individually housed in a temperature and humidity-controlled vivarium that followed a 12-hour light/dark cycle (lights-on at 7:00 a.m.). Animals were provided water ad libitum in their home cage and received a daily ration of high-protein monkey diet (Lab Diet 5045; St. Louis, MO). Additionally, fresh fruits and vegetables were provided as daily enrichment. Experimental sessions were generally carried out 5 days a week (Monday–Friday). All studies were approved by the Institutional Animal Care and Use Committee of the National Institute on Drug Abuse Intramural Research Program.

### Touchscreen apparatus

**Fig. 1:**
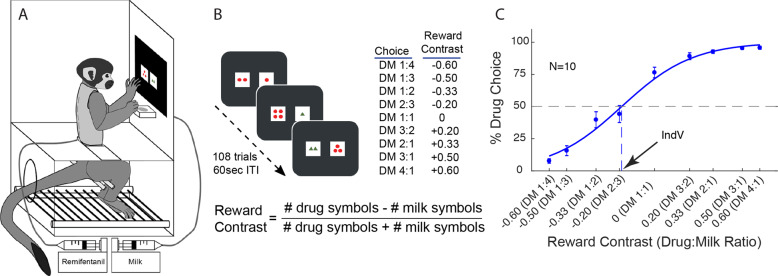
Task design and choice allocation between remifentanil and milk reward. **A** Visual of behavioral apparatus which sits in a sound attenuating chamber. **B** Animals performed a choice task between two stimuli on a touch screen, in which the number of red circles indicated the volume of milk reward and the number of green triangles indicated the amount of intravenous remifentanil reward obtainable by touching the stimulus. The ratio of drug to milk (DM) symbols was used to calculate a reward contrast value as indicated. DM ratios presented to animals and their corresponding reward contrast values are tabulated. **C** Group average of the last 15 baseline weeks for all subjects (*n* = 10). Data points represent the group averages (±SEM) for % drug choice at each reward contrast. The sigmoidal choice curve was fitted by logistic regression of % drug and reward contrast.

During experimental sessions, animals were seated in custom-built acrylic chairs enclosed within ventilated and sound-attenuating chambers (Fig. 1A). A 15 in. touch screen (Elo TouchSystems; Menlo Park, CA) was mounted in a panel directly facing the animal. An external syringe pump (Harvard Apparatus; South Natick, MA) was used to deliver precise volumes of 30% sweetened condensed milk to a drinking well positioned directly beneath the touchscreen. A second external syringe pump was used to deliver intravenous remifentanil hydrochloride dissolved in saline (0.5 µg/ml, see dosing below) during drug self-administration sessions. All programming and data collection for the touchscreen tasks were executed using E-Prime Professional 3.0 (Psychology Software Tools, Inc.; Sharpsburg, PA).

### Touchscreen training

Monkeys were familiarized with the touchscreen with milk reward and taught to choose between two stimuli (two white squares containing 1–4 red circles randomly presented on left or right of the screen midline representing 75 µl/kg milk /symbol). All animals learned to consistently choose the larger reward (>80%). This approach, in which the number of elements is used to indicate the amount of reward, is based on the approach of Padoa–Schioppa and Assad [13].

### Surgery

Following touchscreen training on the milk choice task, subjects were prepared for remifentanil self-administration. Under isoflurane anesthesia and aseptic conditions, a polyvinyl chloride catheter (TYGON^®^; inner diameter, 0.38 mm; outer diameter, 0.89 mm) was implanted in a jugular or femoral vein and exited subcutaneously through the subject’s back as previously reported in greater detail [14]. Animals were allowed to recover for at least one week before beginning training with remifentanil. Monkeys always wore jackets (Lomir Biomedical; Canada) to protect the catheters. Catheters were flushed with saline daily Monday–Friday, and were locked with 0.3 ml of heparin solution (50 USP units/ml; Fresenius Kabi; Canada) on Fridays to maintain patency.

### Remifentanil vs. milk choice task training

For remifentanil reward, a novel stimulus was introduced in which the number of new symbols (1–4 green triangles within a white square) corresponded to intravenous infusion of different unit doses of remifentanil (0.08–0.32 μg/kg/infusion). In initial training experiments, a single milk choice trial was followed by two trials in which a single drug stimulus was presented. A correct touch of the drug stimulus resulted in the infusion of the associated remifentanil dose. Subsequently, single-stimulus drug trials were replaced by drug choice trials, in which subjects chose between two drug stimuli presented simultaneously, one on either side of the screen. Once animals discriminated the larger drug reward (three consecutive sessions >80%), drug choice trials were replaced by drug-milk choice trials in which a drug and a milk stimulus were presented simultaneously (Fig. 1B). The final task consisted of 108 trials, of which 36 were milk vs milk trials (at ratios of 2:3, 1:2, 1:3, and 1:4). Each choice trial was followed by two consecutive remifentanil vs. milk choice trials in order to reduce the potential for drug accumulation and to provide a drug neutral trial type for evaluating the effects of pharmacological treatments. A correct response on either stimulus resulted in delivery of the corresponding reinforcer and initiated an intertrial interval of 60 s.

Each drug vs milk reward choice (Fig. 1B) was randomly presented eight times with stimuli being presented on either side of the screen in a counterbalanced manner. After establishing initial baseline performance, five animals had the unit quantity of milk per symbol doubled (from 0.075 ml/kg/circle to 0.15 ml/circle), in order to increase the symmetry of the choice distribution between the two reward domains. Choice allocation was plotted as the % drug choice vs reward contrast. The reward contrast (Fig. 1B) is defined as the difference in the number of symbols for each of the two offers as a proportion of their sum. This parameterization of choice offers, although frequently used in perceptual studies [15], has not been used in other concurrent drug, nondrug choice procedures. It permits logistic regression to determine indifference values from a single session in which offers of both reward types differ from trial to trial. The choice distribution across the range of offers from milk-dominant reward contrasts on the left side of the x-axis to drug-dominant reward contrasts on the right resulted in a gradual shift in choice from milk reward to drug reward (Fig. 1C).

Indifference values (IndV), defined as the (logistic regression-fitted) reward contrast values resulting in equal choice allocation between drug and milk, have been used previously as a measure of relative preference between rewards [13]. Here, we used it for statistical evaluation of treatment effects (Fig. 1C). In a few cases (17/377 curve fits), when choice data did not follow a full sigmoidal curve (e.g. in a few cases of milk satiation and saline substitution), the calculated IndVs could exceed the natural bounds of the reward contrast values, in which case the IndVs were conservatively estimated at their natural limits (±1; Fig. 1B).

### Testing

All experimental manipulations were initiated on a Monday and maintained throughout the week. Data for either an experimental manipulation or baseline were expressed as an average of the final three days of a given week. Examples of how animals react to treatments on individual days of a treatment week demonstrate that choice allocation shifted quickly (Supplementary Fig S1). Each week-long manipulation was separated by at least one week of responding under baseline conditions in order to provide a contemporaneous baseline comparison.

Experimental manipulations were of two types. In the first type, one of the two primary reinforcers (i.e. remifentanil or milk) was devalued. For milk devaluation, animals were given free access to milk immediately before the session up to a maximum of 60 ml. Typical free-access milk consumption ranged between 30 and 60 ml, compared to ~20 ml of milk chosen during a single baseline choice session. While not an equivalent devaluation manipulation, for remifentanil reward, we assessed reward sensitivity by substituting saline for the drug.

In the second type of experimental manipulation, pharmacological treatments were administered before the initiation of each daily session. Morphine sulphate (NIDA—Baltimore, MD) (1.0 mg/kg) was administered i.m., 15 min prior to the start of the session. Naltrexone (0.1 and 0.3 mg/kg, from Sigma-Aldrich) was administered i.v. 5 min prior to the start of the session via the indwelling catheter.

### Statistical Analysis

All experimental manipulations were statistically compared to the baseline conditions of the preceding week by paired *t*-test (GraphPad Prism 8) with α = 0.05. Different treatments were compared using mixed model ANOVA with Holm-Sidak corrections for multiple comparisons. Response latencies across the different reward contrasts were compared using 1-way repeated measures ANOVA (with Holm-Sidak corrected multiple comparisons). On rare occasions, data from some animals were excluded for a given pharmacological pretreatment. Criterion for exclusion was failure to complete 3 sessions in a treatment week with a minimum of 54 trials within the maximum session duration of 3 h, which was generally interpreted as nonspecific behavioral suppression resulting from sedation. (Minimum time to complete the 108 trials is approx 2 h). Occasionally, an animal missed a treatment week because a catheter needed replacement.

## Results

**Fig. 2:**
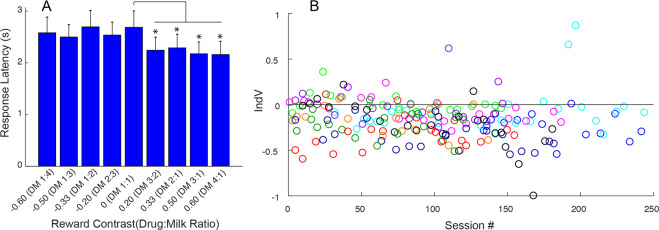
Response latencies and stabilty of choice distribution. **A** Group response latency (over last 15 baseline weeks) varied across different reward contrasts. Post-hoc analysis revealed significant decreases in response latency for all drug-dominant reward contrasts compared to the zero reward contrast (DM 1:1) using Holm Sidak’s multiple comparison correction. *p < 0.01. **B** There was no significant correlation of IndV with cumulative remifentanil exposure (session number). Each of 10 subjects is represented by a different color.

Response latencies were calculated as the difference in time between the onset of the choice stimuli and the touch of the selected stimulus. In order to avoid skewing of the average response times by a few trials during which animals were distracted, average response latencies for each session were caculated after eliminating data points exceeding the 90th percentile of the the response time distribution. The response latencies varied as a function of reward contrast (1-way RM ANOVA (*F*(1.566, 14.10) = 4.82, *p* = 0.032) with higher drug reward contrast choices (more remifentanil) being made significantly faster than choices between a single milk and drug symbols (reward contrast = 0). (Fig. 2A). A summary of response latencies for each treatment and contemporaneous baseline is shown in Supplementary Fig. S1.

Stability of baseline choice behavior over time was determined by examining a partial correlation between IndV of all baseline weeks vs number of sessions, controlling for subject (Fig. 2B). The IndVs for baseline weeks were stable over the many months of testing as indicated by the lack of a significant (partial) correlation between IndV and cumulative dose of remifentanil represented by session number (controlling for between subject differences; *ρ* = −0.0880, *p* = 0.19, Fig. 2B).

### Reward devaluation experiments

**Fig. 3:**
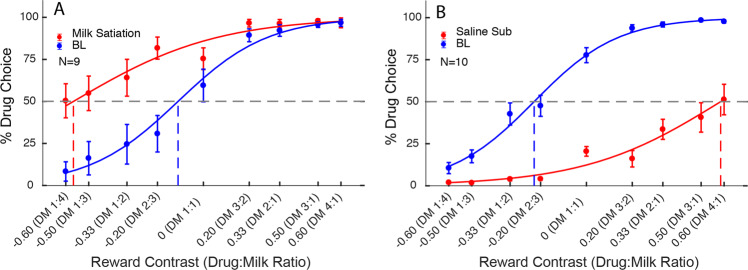
The effects of milk satiation and saline substitution on drug-choice probability. Data points are group means ± SEM. **A** Milk satiation increased drug-choice. **B** Saline substitution (*n* = 10) decreased drug-choice.

**Fig. 4:**
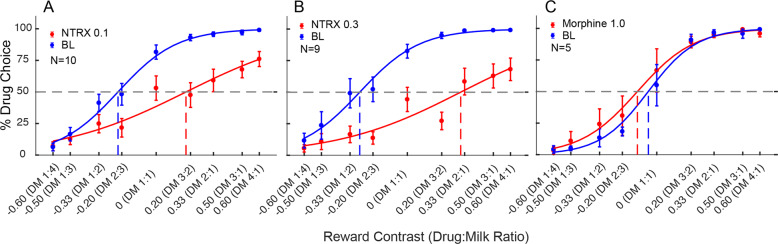
The effects of μ-opioid agonists and antagonists on remifenanil choice. **A** Naltrexone 0.1 mg/kg decreased drug choice, as did **B** Naltrexone 0.3 mg/kg. **C** Morphine 1.0 mg/kg did not affect choice allocation.

Free access to milk prior to the testing session (milk satiation, *n* = 9) significantly increased drug choice probability (Fig. 3A) and decreased IndV (paired *t*-test *t*(8) = 3.877, *p* = 0.0047; see Fig. 4 for summary of all experimental manipulations). The amount of milk consumed during choice was reduced to 50 ± 10% of the preceeding baseline week. It has been reported in some studies in rats that choices for nondrug rewards over drug options appear to be resistant to reward devaluation, with multiple sessions in which devalued nondrug reward was delivered were necessary before a switch in preference was seen [16]. However, we saw this switch in preference even on the first day of presentation of devalued milk (Supplementary Fig. S2). Saline substitution (*n* = 10) decreased choice of the drug stimuli (Fig. 3B), with IndV significantly increased compared to baseline (*t*(9) = 10.31, *p* = 10^−5^).

### Pharmacological pretreatments

We also devalued remifentanil reward by pretreating subjects with an opioid antagonist. Pretreatment with 0.1 mg/kg naltrexone (Fig. 4A), a long acting and potent antagonist [17] resulted in a significant increase in IndV (*t*(9) = 5.365, *p* = 0.0011). Given that pretreatment with 0.1 mg/kg naltrexone did not reduce remifentanil choice as much as saline substitution, the dose was increased to 0.3 mg/kg (Fig. 4B) in order to ensure high μ-opioid receptor occupation. The higher dose of naltrexone produced a greater reduction of remifentanil choice than the 0.1 mg/kg dose, but neither pretreatment dose of naltrexone caused an equivalent increase in IndV as saline substitution. Mixed model ANOVA demonstrated a difference between these treatments (Main effect of treatment *F*(1.655, 14.07) = 18.18, *p* = 0.0002) with the effect of naltrexone 0.3 being >0.1 (Holm-Sidak-corrected *p* = 0.0145) and both naltrexone doses producing a smaller effect than saline substitution (Holm-Sidak-corrected *p* = 0.0024 and 0.0142 for Naltrexone 0.1 and 0.3, respectively). Supplementary Figure S2 demonstrates that the day by day effects of saline substitution and naltrexone pretreatment on choice allocation were seen even on the first session, similar to the milk devaluation.

**Fig. 5:**
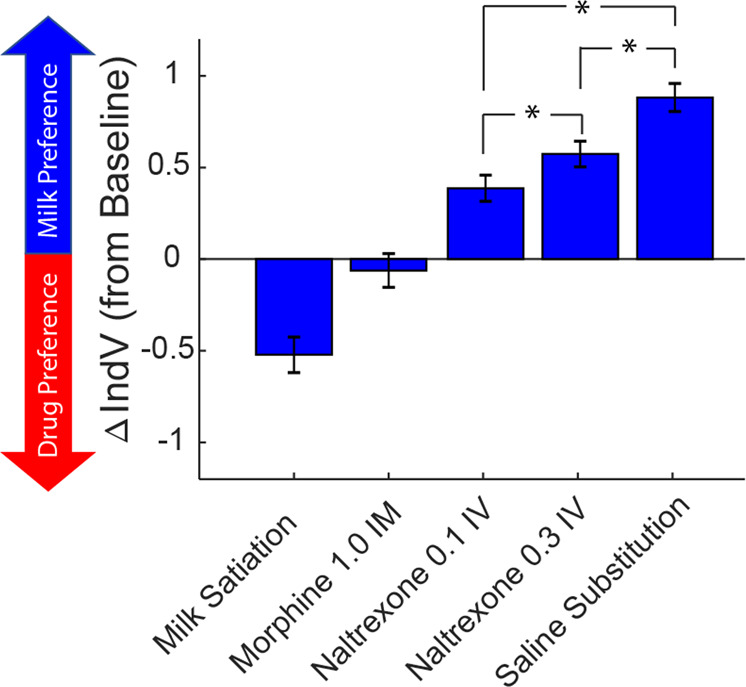
Summary figure illustrating the treatment-induced indifference value change from baseline (∆IndV). A ∆IndV of 0 would indicate no change in drug-choice behavior when compared to baseline. Positive ∆IndV are indicative of decreased drug choice (right curve shift) while negative ∆IndV are indicative of increased drug choice (left curve shift). Saline substitution, naltrexone 0.1 IV, and naltrexone 0.3 IV conditions resulted in a significant positive ∆IndV. Naltrexone effects were different from each other, and both differed from saline. Milk satiation was the only condition that resulted in a significant negative ∆IndV. Data show means ± SEM. *p < 0.05.

We also evaulated the effect of an agonist pretreatment on drug-choice probability, using a 1.0 mg/kg dose of morphine given i.m. 10 min before initiating the choice procedure. (Fig. 4C). There was no significant effect of the agonist pretreatment on remifentanil choice (*t*(4) = 0.6697, *p* = 0.5398). This was a behaviorally active dose as indicated by elongation of response times (Supplementary Fig. S1), and reduced overall responding. Fig 5 summarizes the effects of all treatments on the IndV.

## Discussion

This report summarizes the establishment of a squirrel monkey model of economic choice between the μ-opioid receptor agonist, remifentanil, and a palatable food alternative. Advantages of this model include the use of the very short-acting remifentanil, which cannot accumulate over the session because it is cleared so quickly [9]. This permits a large number of independent trials for each drug-milk choice. Random assignment to left or right across trials also discourages any habitual patterns of responding. Choice behavior was responsive to selective reward devaluation within each reward domain, as measured by shifts in the IndV, demonstrating the economic nature and goal directedness of the choice behavior [18]. This behavioral paradigm, with a broad range of offers between the two domains within a session, provides a sensitive and quantitative framework for examing if new therpautic strategies for addiction treatment can shift behavior away from drug reward toward more naturalistic and beneficial rewards.

The concurrent choice between remifentanil and milk shows a sigmoidal choice allocation curve that shifts from primarily one reward domain to the other as the relative offers of each shift (Fig. 1C). Our indexed value for “relative offers” is a novel metric, the reward contrast, and it accounts not only for the difference in amount between each offer, but also what proportion that difference is of their combined magnitude, similar to a Michelson contrast for comparison of stimuli in studies of visual discrimination [19]. Previous approaches using concurrent drug/nondrug choice procedures have typically examined the change in choice allocation as the unit dose of drug was varied vs a fixed quantity of food [1, 12, 20, 21]. A dose response curve would show a shift away from food choices to drug choices as the unit dose increased, and the visual equivalent of the IndV would represent an ED_50_ for the drug vs a given food offer. While the choice allocation curves we present appear similar, the reward contrast we calculate is different from the unit dose of drug, in that smaller unit doses of drug can be chosen more frequently than larger ones within a given session. For example, on any of the choice allocation curves presented (other than milk satiation), at the DM ratio of 1:1, drug is chosen more frequently than at the ratio DM 2:3, despite offering only half the amount of remifentanil. The subjects are choosing a smaller dose of drug more frequently, because with the higher drug offer, there is an even larger counteroffer of milk, prompting the animal to more often choose the milk option. This is consistent with previous work in the field showing that drug choice depends on the value of the nondrug counteroffer [1, 22–24]. Note that this also demonstrates that animals are not simply choosing on the basis of the number of elements in a stimulus, as might be inferred from the extremes of the choice allocation curve. Animals must instead be evaluating both stimuli for the amounts of potential reward they represent. This establishes that the subjects are considering each offer from trial to trial, without a type of binary responding that can often occur when there is one fixed nondrug offer value vs what is typically a given unit dose of drug within a session, and animals respond only for one reward domain or the other. We do note the work of Negus that established a protocol for within session dose response evaluation for choice between cocaine and food, and that he also demonstrated that session prefeeding of the food alternative shifted choice toward cocaine [22]. However, as noted by Negus, animals in that study tended to respond in a binary manner within a given dose/session block, whereas here, animals shift back and forth from trial to trial, depending on the reward contrast.

By casual observation, squirrel monkeys do not display an equivalent manual dexterity and highly focused attention to touch screen based reward opportunities as macaques. However, response latencies were significantly faster for drug heavy choices compared to milk heavy choices as shown in Fig. 2A. This is consistent with greater motivation for those options, but interestingly this differs from observations with macaques which, counterintuitively, do not show increased response rates for drug offers preferrred over food during concurrent choice [25]. The effect of devaluation on response latencies showed a large increase on milk heavy trials, consistent with these choices being less desired. However, there was also a small increase in latencies on drug heavy choices (Supplementary Fig. S1), and we speculate that this is an additional (familiar) nonspecific effect of a full stomach on response vigor.

The IndV, the calculated reward contrast at which choice allocation is evenly split between the two reward domains, serves as a sensitive, quantitative measure to evaluate alterations in preference between them. This metric remains stable throughout extensive exposure to remifentanil as shown by the lack of correlation between the IndV (from the repeated baseline determinations between manipulations) and cumulative dose of remifentanil (represented by number of sessions) over the course of these studies (Fig. 2B). In this regard, there does not appear to be a differential sensitization over time of the reinforcing effects of remifentanil vs milk. However, withdrawal from more intensive regimens of opioid exposure in physically dependent subjects can produce both somatic withdrawal signs and increased choice of an opioid over a food alternative [3, 26, 27].

We demonstrated the economic nature of choices by selective manipulations of the value of each type of reward. Milk reward was devalued by pre-session satiation. As shown in Fig. 3A, this caused a marked shift in choice allocation toward remifentanil. In order to decrease the value of remifentanil, a number of approaches were used. The first was effectively an extreme dose manipulation by substituting saline for the drug solution. Figure 3B shows a dramatic rightward shift in choice allocation. We believe it noteworthy that even though animals had repeatedly experienced this procedure, and only the data from Wednesday–Friday of each week (with new conditions started on Monday, see Supplementary Fig. S2 for day by day effects) were used, animals still chose “drug” stimulus (actually saline) at the highest drug offers. This is consistent with conditioned effects of the drug cues such that the largest (undelivered) drug offers retained the strongest influence over choice allocation, causing subjects to forego an opportunity for milk reward.

A pharmacological approach was to pretreat subjects with opioid antagonists. We determined the effects of two doses of naltrexone which have previously been shown to effectively block the μ-opioid receptor and the ability of agonists to support behavior [17]. Both doses resulted in reduced drug choice, though less than saline substitution. There are effects of opioid antagonists on nondrug, palatable food reward that suggest they may also be modulating the rewarding properties of the sweetened milk which could affect choice allocation. Specifically, opioid antagonists have been shown to decrease the palatability of sweet tasting solutions, intake of highly palatable foods, and motivation to seek highly palatable foods and sucrose solutions [28–33]. There were no consistent effects of the antagonist on either response latencies or choice for larger reward on the milk-milk trials that might suggest reduced palatibility, and so another potential effect to consider is a brief surmounting of the antagonist by peak plasma concentrations of infused remifentanil, an effect that saline could not produce, thereby creating the distinction.

We also evaluated an agonist pretreament on choice allocation, but 1 mg/kg morphine, did not alter choice allocation. This is consistent with previous work demonstrating that agonists affect choice only in a “dependent” model, consistent with negative reinforcement effects [3]. The most clinically relevant model for the development of treatments for those actively using significant amounts of opiates should therefore include some degree of dependence.

Given the validation of the economic nature of choice behavior this paradigm displays, it will be ideal for future systems neuroscience investigations to determine whether there are distinct neural circuits mediating opioid and food reward. Work has previously been done in rats delineating these mechanisms with cocaine reward [34, 35] and this approach has been extended to opioid vs natural reward [36]. It also will be an ideal model for evaluating the use of contemporary powerful synthetic receptor/molecular approaches such as opsin or chemogenetic methods that could be utilized for targeting distinct opioid/food reward systems.

In conclusion, we have developed an economic choice paradigm that enables comparing indifference points between reward domains using a unique parameter for opioid vs food offers over a wide range of choice options in nonhuman primates. By using squirrel monkeys rather than macaques, larger sample sizes suitable for population comparisons of treatment efficacy will be possible. This advantage will support use of nonhuman primates for investigation of novel pharmacological treatments with high throughput to support exploration of modern opsin and chemogenetic approaches to further our understanding of the mechanisms underlying drug choice, with quantitative metrics for evaluating the impact of targeted, circuit specific manipulations.

## Funding and disclosure

This research was supported by the Intramural Research Program of the NIH, NIDA. None of the authors have competing financial interests.

## Supplementary information

Supplemental Material
