# Supplementary material for: Economic choice between remifentanil and food in squirrel monkeys
Source: Neuropsychopharmacology. 2021 Apr 8;47(7):1398–404. doi: 10.1038/s41386-021-00996-6 (PMC9117236; doi:10.1038/s41386-021-00996-6)
Supplement: Supplementary file 1 — Supplemental Material [file 41386_2021_996_MOESM1_ESM.pdf]

SUPPLEMENTAL MATERIAL

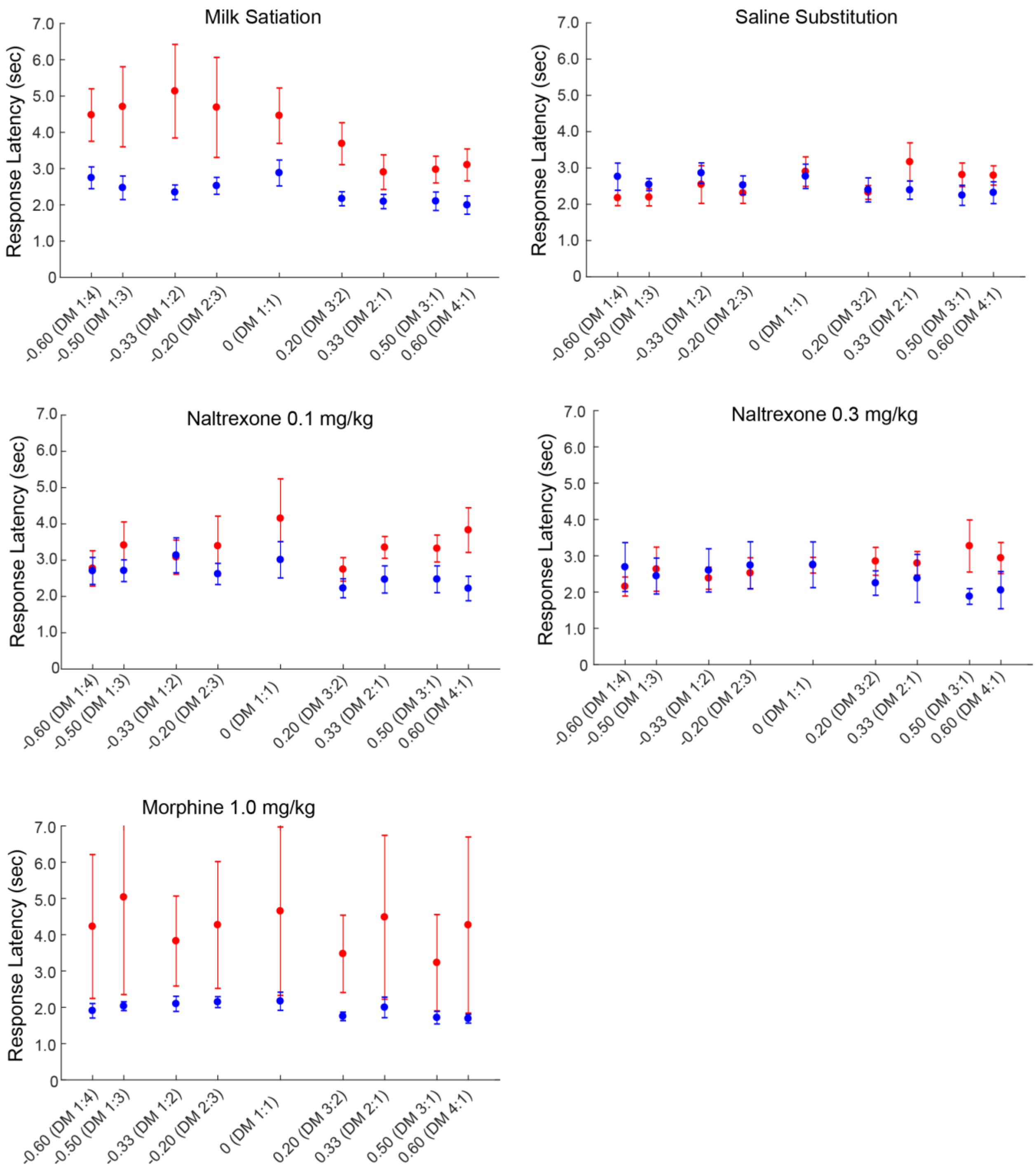

Fig.S1. Response latencies vs Reward Contrast for each treatment and contemporaneous baseline.

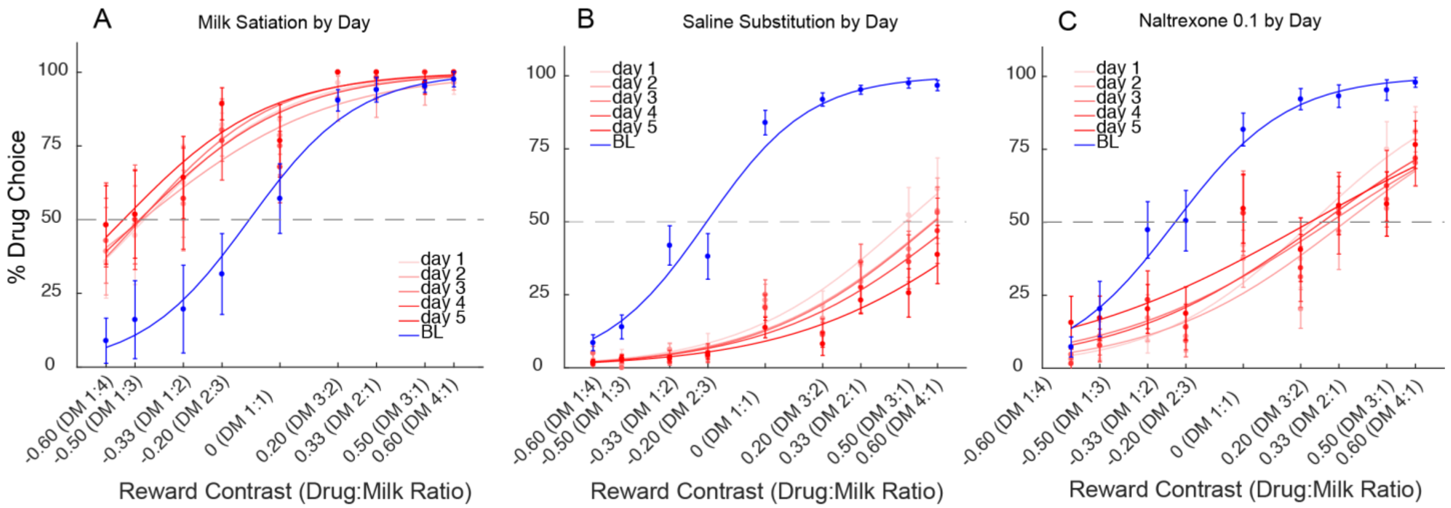

Fig.S2. Representative day by day treatment effects on choice. Treatments were Monday-Friday, with day 1 being Monday. **A)** Milk satiation; **B)** Saline substitution; **C)** Naltrexone pretreatment.
